# Quantifying fluorescent nanoparticle uptake in mammalian cells using a plate reader

**DOI:** 10.1038/s41598-022-24480-3

**Published:** 2022-11-23

**Authors:** Hye Ji Shin, Minjeong Kwak, Sihwa Joo, Ji Youn Lee

**Affiliations:** 1 Biometrology Group, Division of Chemical and Biological Metrology, Korea Research Institute of Standards and Science, 267 Gajeong-Ro, Yuseong-Gu, Daejeon, 34113 Republic of Korea; 2 Nanosafety Team, Safety Measurement Institute, Korea Research Institute of Standards and Science, 267 Gajeong-Ro, Yuseong-Gu, Daejeon, 34113 Republic of Korea; 3 Bioimaging Team, Safety Measurement Institute, Korea Research Institute of Standards and Science, 267 Gajeong-Ro, Yuseong-Gu, Daejeon, 34113 Republic of Korea

**Keywords:** Biotechnology, Analytical biochemistry

## Abstract

In keeping with the rapid expansion of nanoparticle applications, various tools are required to investigate how nanoparticles interact with biological entities. Many assays have been developed to measure the cellular uptake of nanoparticles, but so far most of the methods are laborious and often non-quantitative. Here we developed an easily accessible and robust quantitative measurement method of the level of cellular uptake of fluorescently labeled nanoparticles using a plate reader. In the experimental design, potential issues that could lead to measurement variation were identified and addressed. For example, the variation in fluorescence intensity of samples due to differences in cell number was normalized to optical density, which is a physical value corresponding to the cell number. Number of washings and sample handling temperature were optimized to minimize the interference by residual nanoparticles and possible efflux of nanoparticles from cells, respectively. The developed assay was demonstrated with the lymphocyte cell line Jurkat to measure the cellular uptake of fluorescently labeled 50 nm polystyrene beads, and its applicability was further confirmed with the lung carcinoma cell line A549 and another lymphocyte cell line RPMI8226.

## Introduction

Recent developments in nanotechnology provide diverse opportunities in healthcare^1^, foods^2^, and cosmetics^3^ industries, while they also raise concerns about potential toxicity^4,5^. In order to investigate the performance and safety of nanomaterials, we need to understand their lifecycle consisting of encounter and recognition – internalization – trafficking – distribution – and efflux via various biological experiments. Nanoparticle uptake is a broad process including encounter, recognition, and internalization^6^. Depending on the usage, the level of uptake needs to be modulated either to increase the delivery or to avoid indiscriminate uptake. For example, in medical applications, while nanoparticle clearance by macrophages should be avoided^7^, nanoparticle delivery efficiency to a target organ should be improved. A wide range of nanomaterial uptake studies have been performed with mammalian cells^7–9^, prokaryotes^10^, and plants^11^, to name a few. Cellular uptake is governed by many different parameters^6,12^ such as the physical properties of nanoparticles including size, shape, and surface charge, as well as the experimental conditions including cell types^13^ and cell cycle^14^. Even cell-to-cell variability exists in nanoparticle uptake, as revealed in a sophisticated experiment by Åberg et al.^15^.

Numerous analyses have been employed to quantify the uptake of nanomaterials^16^. The most commonly used are techniques based on electron^17–19^ or light microscopy^20,21^. Measurements of optical properties such as scattering and fluorescence have also been employed for quantification with flow cytometers^8,10,14^ and microplate readers^19,22–27^. Inductively coupled plasma (ICP)-based techniques, such as ICP-mass spectrometry^28^ and ICP-atomic emission spectroscopy^26,29^ are another useful tool to quantify nanoparticles in cells, but they are destructive and only applicable to metal particles. Other techniques, such as transmission X-ray microscopy, whole cell tomography, and hyperspectral imaging, have been adopted as well, but their applications are limited. Although a number of techniques for nanoparticle quantification in cells and tissues exist, more reliable, reproducible, and intercomparable techniques, that are also commonly used, are still in demand^30^.

Plate readers are found in wide measurement applications ranging from as small as metal ions^31^ to as big as cell spheroids. Their multimodal and high-throughput capabilities have facilitated the development of diverse cell-based analyses including proliferation, death, enzyme activity, and other phenotypic assays. Combination with recent advances in liquid handling and environment control systems has enabled more sophisticated dynamic analyses. Above all, plate readers are easy-to-operate equipment not requiring high skill that can be used in a variety of ways.

Among the commonly measured optical properties, fluorescence is often used in biological assays because it enables the analysis of biological molecules even at low concentrations, and it also provides multiple options in selecting particular fluorescent molecules depending on their wavelengths. Fluorescent molecules conjugated to nanoparticles serve as a reporter to investigate the location and functions of the particles. In our previous work^32^, we developed a nanoparticle uptake assay using flow cytometry, with which we quantified the level of fluorescently labeled silica nanoparticle uptake by measuring the fluorescence intensity of a cell population. In this assay, the intensity can be calibrated to molecules of equivalent soluble fluorophore for a comparison between different sets of experiments.

Plate readers have been employed to quantify the cellular uptake of various nanoparticles, such as polystyrene^9,23^, silver^19^, chitosan^24^, silica^25^, and quantum dots^26^. For quantification, the amount of nanoparticles per cell number^9,23^ or amount of protein^24,25^ and the uptake relative to control samples^19,26^ were often found. Most assays used lysed cells rather than washed cells, making further downstream analysis difficult. These published works show that the microplate reader is a useful tool for nanoparticle uptake assays, but in many cases, investigations into experimental details and the reliability of the methods have been lacking.

In the current work, we focus on developing a robust and reliable yet easily accessible nanoparticle uptake assay without cell lysis using a plate reader. For this purpose, we first anticipated potential issues that may appear in the experimental procedure, and then specifically designed the assay to address such issues. We then implemented the method with a human lymphocyte cell line, Jurkat, and fluorescently labeled polystyrene nanoparticles, and cross-examined with our previously developed assay based on a flow cytometer. Finally, we applied the assay to an adherent cell line, A549, as well as to another suspension cell line, RPMI8226, for further validation.

## Results and discussion

### Experimental design: considerations

**Figure 1:**
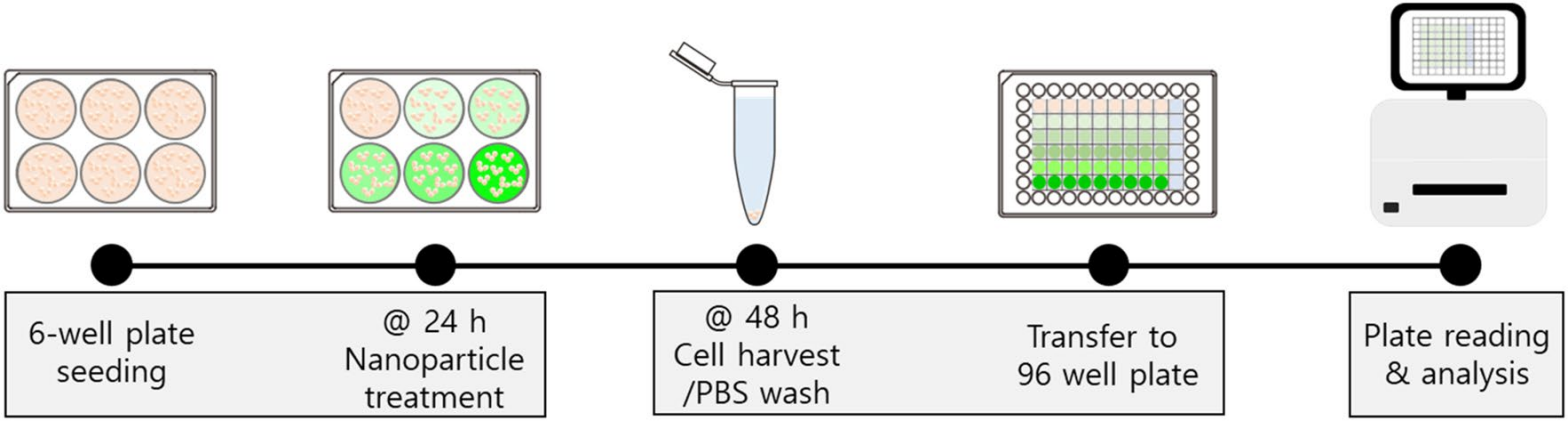
Overall experimental procedure for nanoparticle uptake analysis including cell seeding, nanoparticle treatment, cell harvesting and washing, and plate reading and analysis.

In this work, we aimed to develop a reliable, robust, and also easily accessible assay for quantifying fluorescent nanoparticle uptake in mammalian cells using a plate reader. For this purpose, we tried to first identify key factors that would affect the readout values and investigate them to achieve reliable measurement. Figure 1 summarizes the experimental procedure of the developed assay. The assay largely consists of three steps: (1) culturing cells and treating with fluorescent nanoparticles in a 6-well plate, (2) washing the nanoparticle-treated cells and aliquoting the washed cell solution in a 96-well plate, and (3) reading the fluorescence intensity of the cell solution using a plate reader and analyzing the results.

The first we identified affecting the reliability of the measurement results is the difference between cell numbers among the biological and technical triplicate samples in the 96-well plate largely arising from cell handling. The second is the possible presence of nanoparticles in the cell solution by insufficient washing. Residual nanoparticles lead to a bias in the measured fluorescence intensity that would be directly considered as the level of nanoparticle uptake. The third significant factor is the loss of the fluorescence signal from the nanoparticle-treated cells due to the efflux of nanoparticles when the cell culture medium containing the nanoparticles is replaced with a wash buffer. To resolve these issues and therefore make the assay more robust, we devised a reasonable solution to each that will be presented in detail in the following sections. With our developed assay, we applied it to three mammalian cell lines—two blood cell lines, Jurkat and RPMI8226, and one lung cell line, A549. We will first present the experimental results of the Jurkat cells, which were mainly used to develop the assay, and then the results of the A549 and RPMI8226 cells.

As a model nanoparticle, we choose 50 nm internally dyed polystyrene nanoparticles, PSNP50. Their nominal size and average z-diameter in basal and complete culture media measured by dynamic light scattering (DLS) were 50, 46.23, and 83.63 nm, respectively (supplementary data, Fig. S1). The average diameter determined by scanning transmission electron microscopy (STEM) was 40.07 nm. The difference in particle size measured by DLS and EM, about 6 nm, is due to the wide distribution of particle size. As shown in the STEM image and size distribution graph, a significant number of nanoparticles (approx. 10%) smaller than 30 nm were present. The zeta potential of the nanoparticles was measured as − 47.30 mV and − 13.50 mV in basal and complete culture media, respectively.

The fluorescence intensity (FI) of PSNP50 was analyzed via total internal reflection (TIRF) microscopy. The FI and its distribution was compared with those of in-house synthesized FITC-labeled silica nanoparticles (SiO_2_NP) of different sizes that were synthesized as previously described^32^. The average FI of the PSNPs was lower than that of SiO_2_NPs of similar size; however, the FI of the PSNPs showed a narrower distribution compared to SiO_2_NPs, suggesting that PSNPs are suitable for quantitative nanoparticle uptake analysis (supplementary data, Fig. S2).

### Investigating the relationship between cell number and optical density in Jurkat cells

In our assay, we measure the integrated fluorescence intensity from an ensemble of cells in the well, and the intensity is in turn assessed as the level of nanoparticle uptake. Ideally, the same number of cells is seeded in each well and the same sample handling is performed for each well; however, in reality, the number of cells between wells in a 96-well plate for fluorescence intensity reading can vary. Sources of variation include pipetting and the recovery efficiency in centrifugation that are essential steps in cell harvesting and washing. If the number of cells between different wells varies too much, the resulting uptake assay will be unreliable. Therefore, it is desirable to normalize the fluorescence intensity with cell number in the analysis step. While cell counting with counting devices might be the best option, counting cells in several tens of samples is time-consuming and not easily achievable. Also, the cell number in individual wells may not be high enough for most cell counters to provide reliable measurement. Other high-throughput microplate-based colorimetric assays for cell number estimation such as MTT assays are not compatible with our assay due to interference from the fluorescent nanoparticles. Therefore, in this work, we used the optical density (OD) of the cell solution as a cell concentration surrogate to normalize the fluorescence intensity.

While OD measurement has been commonly used to monitor bacterial cell growth^33^, it has rarely been employed in mammalian cell cultures^34,35^. Instead, related studies routinely apply direct cell counting using a hemocytometer or automatic cell counter, or indirect cell counting methods that measure the metabolic activities of cells or the DNA amount of cell pellets. Measurements of OD have been mainly limited by the many components in the culture medium affecting the optical properties of the cell solution. However, in our assay, the culture medium is almost completely removed during the washing step and the cells are resuspended in 1 × PBS, which allows us to expect that OD will be highly correlated with cell concentration.

**Figure 2:**
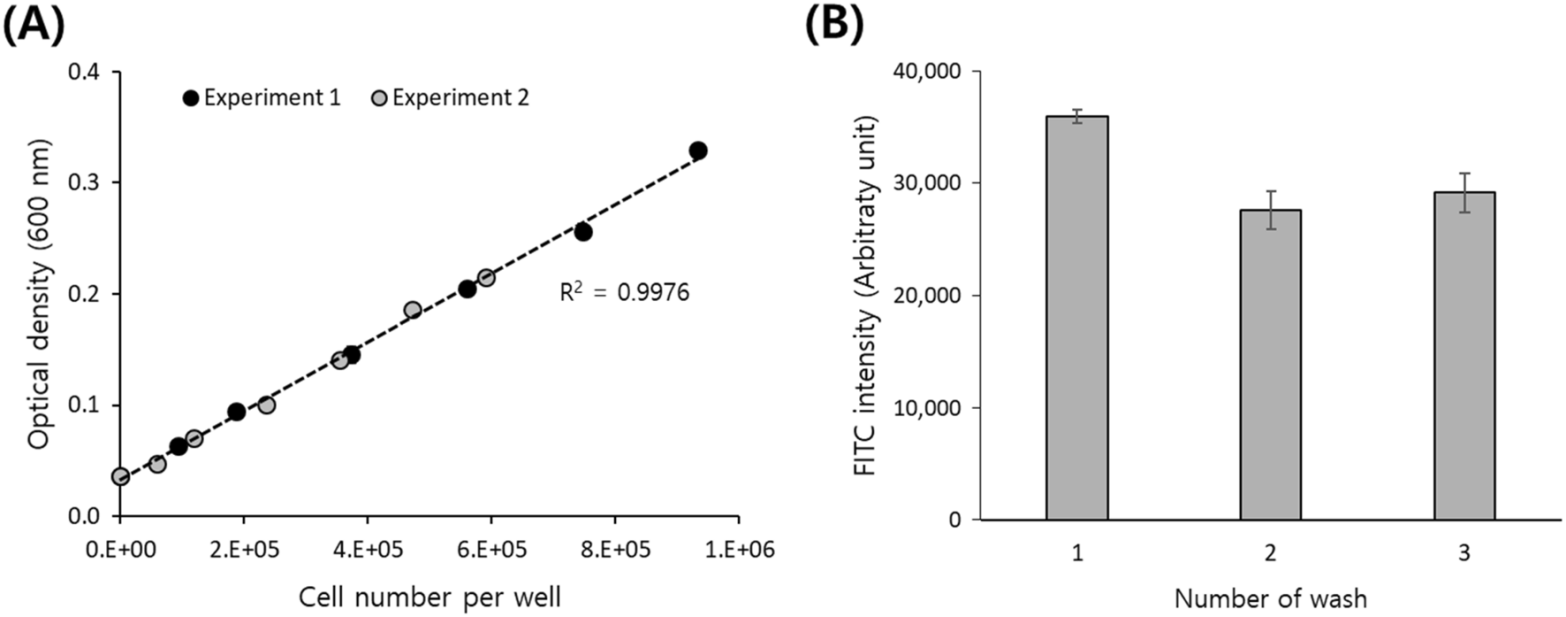
(**A**) Correlation between cell number and optical density measured at 600 nm and (**B**) FITC intensity change by repeated washing of Jurkat cells.

First, we checked whether the nanoparticles can affect the OD of the cell solution. The presence of nanoparticles lower than 10 µg/mL does not contribute to OD regardless of the wavelength. In the case of 100 µg/mL, the optical density of the nanoparticles was not negligible and more pronounced at shorter wavelengths due to the green dye embedded in nanoparticles (supplementary data, Fig. S3). Then we obtained a plot between cell number and OD to see the correlation. Serial dilutions of Jurkat cells ranging from 0.59 to 9.3 × 10^5^ cells per well in a 96-well plate were prepared, and the ODs were measured at various wavelengths ranging from 400 to 650 nm with 50 nm intervals. For precise cell counting, we used counting beads and flow cytometry instead of an automatic cell counter. The gating strategy is summarized in the supplementary data Fig. S4(A). The plot between cell number and OD shows a fairly linear relationship at 600 nm with R^2^ values higher than 0.99, as shown in Fig. 2A. The measurement was reproducible, and the slopes from two separate experiments (grey and black circles in Fig. 2A) were almost identical. Similar results were obtained for ODs obtained at other wavelengths. Taken together, we can conclude that OD can be used as a surrogate parameter for cell number for the normalization of the fluorescence intensity, thus compensating for deviations in cell number between wells. In all tested wavelengths from 400 to 650 nm, the effect of the nanoparticles on OD was not significant in the measured range, and the linearity of cell concentration and OD was good enough (Supplementary data Fig. S5). Therefore, in subsequent experiments, 600 nm, which is less affected by the green dye used in this work than shorter wavelengths and is commonly used to measure the concentration of microbial cells, was selected for analysis. Shorter wavelengths can be selected for normalization to avoid potential interference from the dye molecules, when using nanoparticles containing dyes of longer wavelengths.

### Washing test of nanoparticle-treated Jurkat cells

As explained in the experimental design section, residual nanoparticles in the cell solution can affect the fluorescence reading by a plate reader. Multiple washings of the cell solution assure the complete removal of nanoparticles; however, adverse effects from prolonged exposure of cells to serum-free and nutrition-free environments and the possible efflux of nanoparticles from cells to the wash buffer can occur. Therefore, rather than small volume washings, we used a large volume of wash buffer, namely 5 mL per wash, for cells from one well of a 6-well plate containing approximately 5 × 10^5^ cells. Then to optimize the number of washings, cells washed a different number of times were subjected to fluorescence measurements using a flow cytometer. For cells washed twice, a large decrease in the FITC signal was observed compared to that after one washing, but no noticeable decrease in the FITC signal was observed after a third washing (Fig. 2B). So, we concluded that two washings were sufficient to remove the nanoparticles from the culture medium and accordingly give reliable plate reader results. In addition, we observed a gradual decrease in FITC intensity when cells were left in 1 × PBS at room temperature, possibly due to nanoparticle efflux from the cells to the buffer. It has been reported that nanoparticle uptake is temperature-dependent, where 4 °C allows only binding of nanoparticles to the cell membrane and inhibits internalization^36^. Therefore, sample handling was performed on ice as much as possible to prevent fluorescence signal loss.

### Analysis of nanoparticle uptake in Jurkat cells using a plate reader

Jurkat cells in a 6-well plate were treated with nanoparticles ranging from 5 to 100 µg/mL (5, 12.5, 25, 50, and 100) for 24 h. First, we investigated if the presence of nanoparticles affects either the cell morphology, viability, or proliferation. As shown in Fig. S6, cell morphology was checked under a microscope and no noticeable changes were observed. High cell viability was maintained, and the number of cells treated with different concentrations of nanoparticles was measured to be similar regardless of the nanoparticle treatment. After the treatment, we prepared biological and technical triplicate samples in a 96-well plate as described in the experimental schematic (Fig. 1). Our sample layout in the 96-well plate for plate reading is presented in Fig. S7.

**Figure 3:**
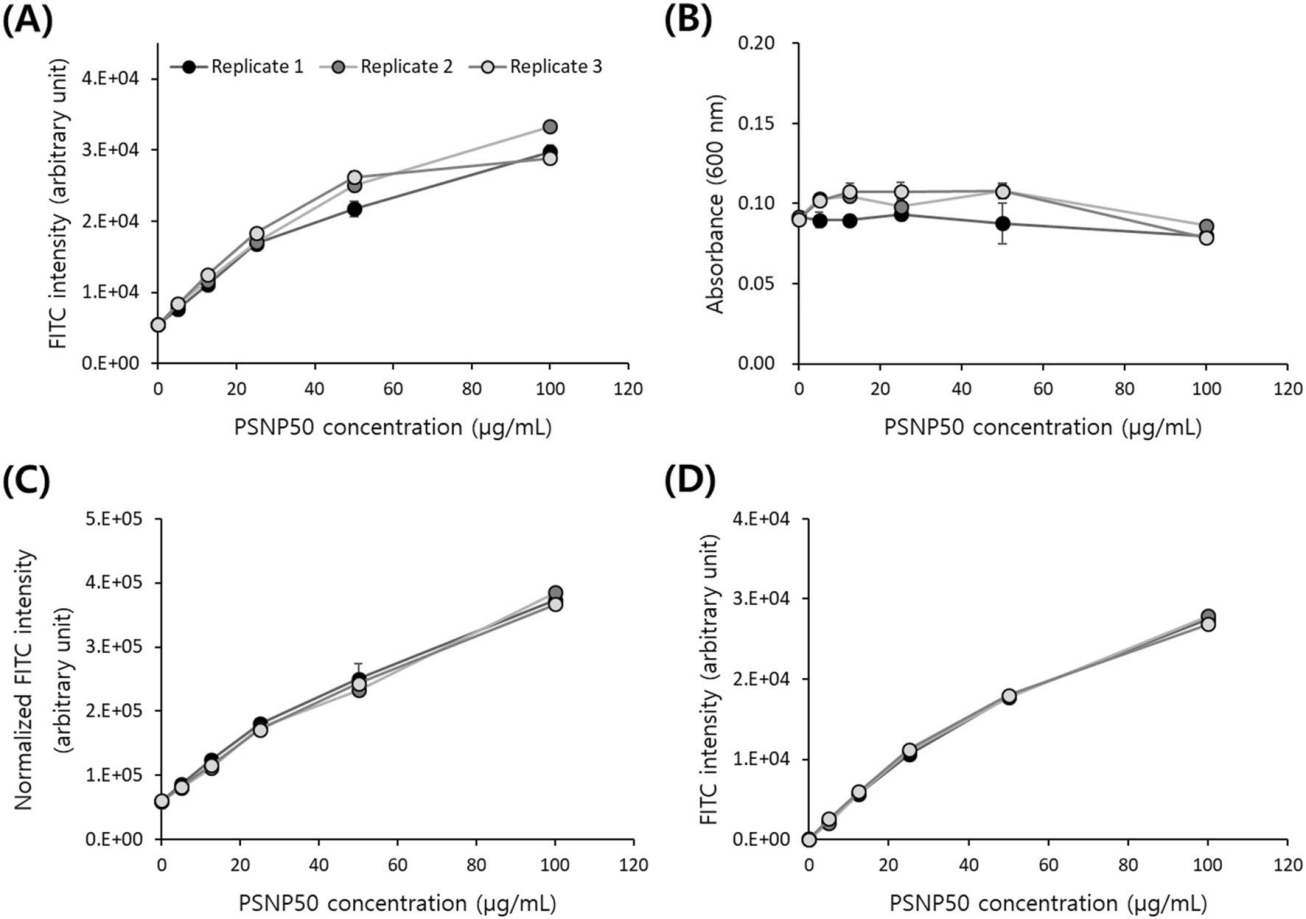
Quantification of nanoparticle uptake by Jurkat cells for biological replicates. The scatter plots show three biological replicates of Jurkat cells treated with different concentrations of PSNP50. (**A**) FITC intensity measured by a plate reader, (**B**) optical density measured at 600 nm by a plate reader, (**C**) normalized FITC intensity by the optical density, and (**D**) FITC intensity measured by a flow cytometer.

As shown in Fig. 3A, the FITC intensity of the Jurkat cell solution, measured with a plate reader, increased with increasing concentration of treated nanoparticles. Although the three biological replicates showed similar trends, there was variation between them, and the CV was up to 9.41% for the 50 µg/mL sample. We normalized the FITC intensity to the OD value of the same well measured at 600 nm (Fig. 3B). After normalization, the variation between the three replicate samples was significantly reduced, as shown in Fig. 3C, where the CV decreased to 3.63% for the 50 µg/mL sample. A decrease in CV was observed in all nanoparticle concentrations, and was particularly evident in the higher concentration samples. The corresponding raw and analyzed data for this set of experiments are summarized in the supplementary data Table S1. For a cross-validation, we also performed the flow cytometer-based nanoparticle uptake assay developed in our previous work^32^. Live single Jurkat cells were gated according to the strategy outlined in supplementary data Fig. S4(B), and the median FITC intensity was plotted. As shown in Fig. 3D, the flow cytometer measurement showed a very similar trend to our plate reader measurement. Considering that the flow cytometer-based assay measures the FITC intensity of individual cells, we can indirectly confirm that the normalization of FITC values using OD corrects the differences in cell numbers.

Then we investigated the uptake kinetics of Jurkat cells. They showed an immediate uptake of PSNPs upon nanoparticle exposure. The normalized FITC intensity of cells that were exposed to nanoparticles for 2 h was 70% of that measured from cells treated for 24 h (supplementary data, Fig. S9). In our previous work, we similarly observed that THP-1 cells, a monocyte in suspension culture, showed an immediate uptake of silica nanoparticles, while adherent-type cells—A549, HCC827, and HaCaT—showed a steady linear increase of nanoparticle uptake for 24 h^32^. Also, a similar instant uptake of polyisoprene nanoparticles by Jurkat cells has been previously reported^37^.

**Figure 4:**
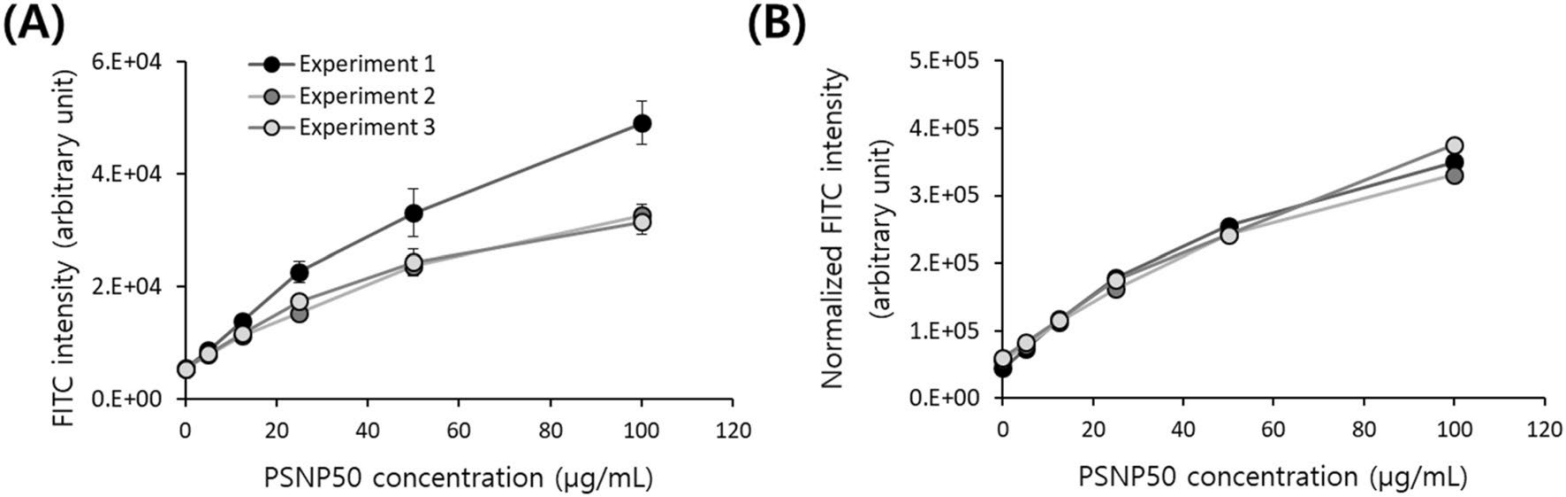
Quantification of nanoparticle uptake by Jurkat cells in multiple experiments. The scatter plots show three different sets of experiments with Jurkat cells treated with different concentrations of PSNP50. (**A**) Before and (**B**) after the normalization of FITC intensity with absorbance at 600 nm.

To further validate the assay, three independent sets of experiments were performed on different days and compared. Although we followed the same protocol for each assay, the FITC measurements in one of the experiments (Experiment 1) were different from the others, as shown in Fig. 4A. Measurement data is likely to fluctuate due to various factors such as differences in cell number caused by differences in proliferation according to the passage numbers or the initial cell seeding density, and differences in cell loss during cell harvesting and washing. If the fluctuations are due to differences in cell number, they can be corrected by normalizing the FITC intensity to OD. Indeed, the normalization significantly improved the level of agreement between experiments as presented in Fig. 4B; the CV change by normalization is shown in Fig. S10.

The correlation between nanoparticle concentration and fluorescence intensity was high, especially in the low concentration range as shown in Fig. S11. Using the linear relationship, we can calculate the minimum concentration of PSNP50 required for uptake quantification. In our experimental setup, A549 cells treated with as low as 1 µg/mL of PSNP50 could be detected, based on the background fluorescence intensity of the untreated negative control.

### Analysis of nanoparticle uptake in A549 and RPMI8226 cells using a plate reader

**Figure 5:**
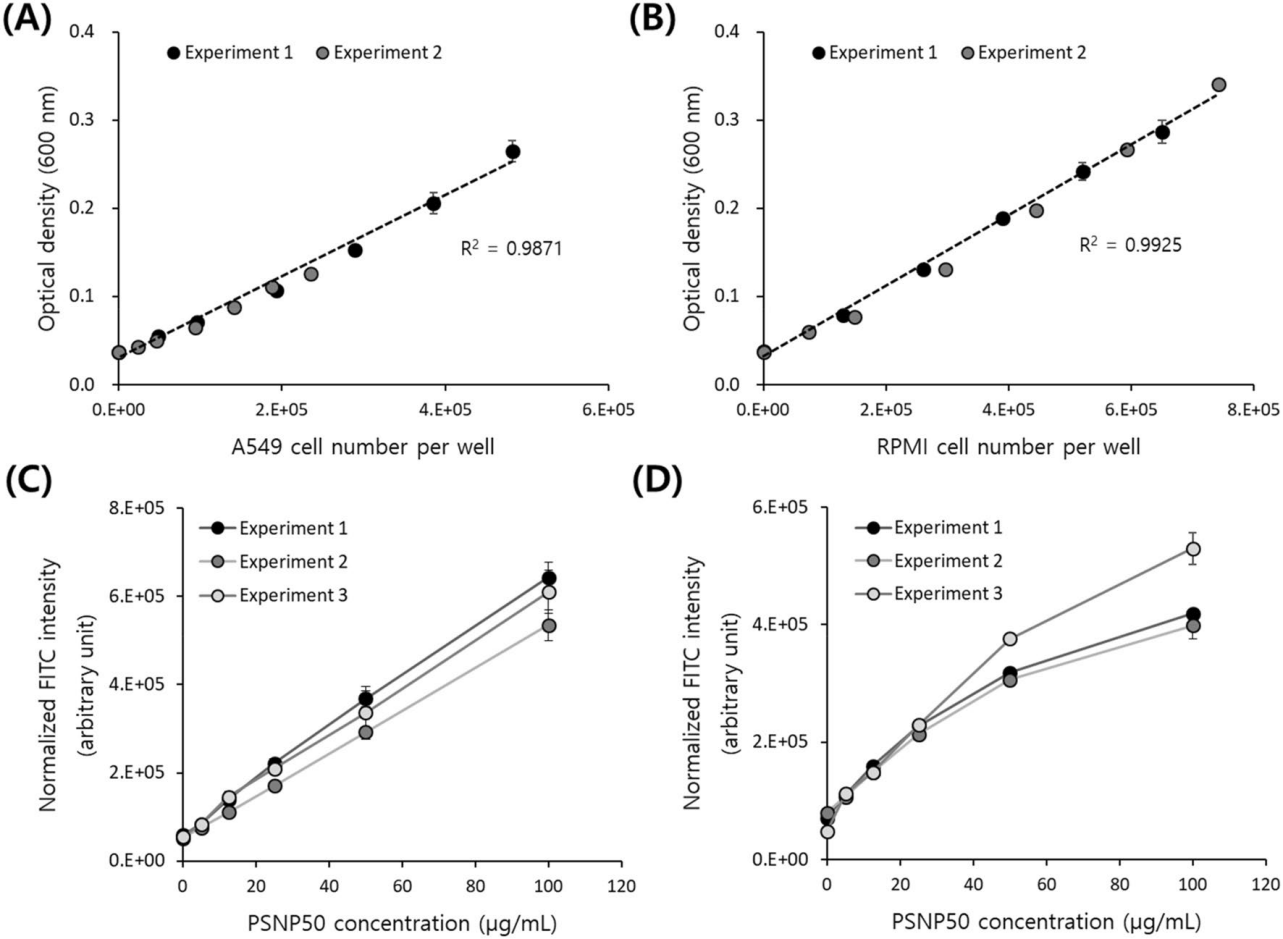
Scatter plots showing the correlation between cell number and optical density measured at 600 nm for (**A**) A549 and (**B**) RPMI8226 cells. FITC-PSNP50 uptake results of different sets of experiments with (**C**) A549 and (**D**) RPMI8226 cells.

To investigate the versatility of the developed assay, we further tested two other types of cells. One is the adherent cell line A549, a human lung carcinoma cell, and the other is a second suspension cell line, RPMI8226, a human B lymphocyte. First, for both cell lines, the correlation between cell number and OD was investigated, with the results presented in Fig. 5A and B. For the A549 cells, two separate experiments with cell numbers ranging from 0.42 to 7.4 × 10^5^ cells per well in a 96-well plate showed a good correlation between cell number and OD. Likewise, the RPMI8226 cells ranging from 0.4 to 7.5 × 10^5^ cells per well in a 96-well plate showed a good linear correlation between cell number and OD as well. All three cell lines showed a linear relationship between cell number and OD, but with different coefficients (i.e., slopes). The contribution of cells to the signal intensity of OD was highest in A549 and lowest in Jurkat cells (supplementary data Fig. S12(A)). The optical density of cells depends on their size and complexity. We therefore looked at the scattering parameters obtained from a flow cytometer, namely the forward scatter (FSC) and side scatter (SSC) that reflect the size and the complexity of an object, respectively. As shown in supplementary data Fig. S12(B) and S12(C), both parameters were the highest in A549 and lowest in Jurkat, and these results explain the variation in the slope of the correlation between cell number and OD among the cell lines.

Then we investigated whether the nanoparticle treatment affects the cell morphology and proliferation of the A549 and RPMI8226 cells, as we observed in the Jurkat cells. Regarding cell morphology, no noticeable change occurred in either cell line for all treated nanoparticles (Fig. S13(A) and S13(B)). For the A549 cells, cell proliferation was measured via live imaging followed by cell count analysis. Nanoparticle-treated cells showed a similar growth pattern regardless of the nanoparticle concentration, demonstrating that the presence of nanoparticles up to 100 µM did not affect cell proliferation (Fig. S13(C)). Prolonged culture for 72 h also showed no significant nanoparticle concentration-dependent change in cell growth (Fig. S13(D)). For the RPMI8226 cells, the cell number that was precisely estimated by a flow cytometer using counting beads was maintained at a constant level regardless of the concentration of treated nanoparticles (Fig. S13(E)).

Then nanoparticle uptake was analyzed for both cell lines using the developed assay. We could observe similar nanoparticle concentration-dependent uptake results in both cell lines, similar to those observed in Jurkat cells. RPMI8226 showed more similar results to Jurkat cells, exhibiting a rather linear increase of FITC intensity up to 25 µg/mL followed by a slightly retarded increase at higher concentrations, whereas A549 cells showed a fairly linear and consistent increase in FITC intensity with increasing concentration of treated nanoparticles, as shown in Fig. 5C and D. We could reconfirm that normalization using the OD reduced the deviation between biological replicates of the cells by comparing scatter plots before and after the normalization, and also by the flow cytometer results, as presented in supplementary data Fig. S14 and S15.

For samples treated at lower nanoparticle concentrations, normalization by OD did not necessarily improve the CV values. Since each well of the 96-well plate contains a similar number of cells, the OD of each well (i.e., the denominator for the normalization) remains constant regardless of the treated nanoparticle concentration, whereas the fluorescence intensity is highly dependent on the concentration. For samples treated with 5 µg/mL nanoparticles, the fluorescence intensity is very low, close to the background level, which can lead to fluctuations in normalized values. However, for samples of FITC intensity higher than the background level—in our assay, cells treated with 10 µg/mL or higher concentrations of nanoparticles—it was confirmed that the CV could be significantly lowered, and the reliability of the measurement could be improved.

### Nanoparticle uptake assay with A549 cells cultured in a 96-well plate

Lastly, we considered the case of using a single 96-well plate for the entire assay, in other words removing the harvesting and transferring steps. If we use the adhered cells as-is without transfer for the fluorescence reading, measurement fluctuations caused by transferring the cell solution from the 6-well plate to the 96-well plate and washing the cells by centrifugation are eliminated. As long as washing the adherent cells in the wells effectively removes the nanoparticles and the fluorescence signal is high enough to quantify the level of uptake, we expect the measurement results from this format may also be reliable. For this, we investigated whether the measurement results from the developed assay are comparable to the case in which both cell culture and fluorescence reading are performed in the same well plate for adherent cells.

In this format, the adherent A549 cells were first cultured in a 96-well plate, treated with nanoparticles, and then washed carefully. Fluorescence was measured with a plate reader. We could observe a nanoparticle dose-dependent linear increase in FITC intensity of treated A549 cells, a trend very similar to the results observed with A549 cells in suspension (Fig. S16(A)). However, in this format, there were technical difficulties in the washing step to remove residual nanoparticles. We need to wash cells gently by pipetting to prevent cell detachment; however, even with careful washing, some cells started to fall off after the second washing. The fluorescence intensity decreased after the second washing (Fig. S16(B)), but it was difficult to determine the cause among the removal of residual nanoparticles, the unavoidable detachment of a small number of cells during the washing, and the nanoparticle efflux from cells during the washing because no further washing could be carried out due to the cell loss. Although we could show that, for adherent cells, the nanoparticle uptake assay can proceed with a single 96-well plate for both cell culture and fluorescence measurement, further optimization and validation are required for a reliable assay.

In this work, we developed a microplate-based fluorescently labeled nanoparticle uptake assay that can be used for both suspension and adherent cells, and successfully applied it to three mammalian cell lines. During the development, we considered possible factors that could affect the readout and explored ways to increase the reliability of the assay. As a result, the fluorescence intensity was normalized by the optical density that largely corresponds to the cell number, which improved the agreement between the biological and technical replicates, and also between multiple sets of experiments run on different days. Considering that the experimental results are greatly affected by the cell status depending on passages or culture conditions for cell-based assays, normalizing measurement values by optical density, that is directly related to the cell number, is a beneficial step for the developed assay. This is especially useful because only additional reading in absorption is required when reading the fluorescence without additional experimental procedures. In addition to the normalization, the number of washings and sample handling temperature were optimized to provide reliable results. Further optimization, such as using phenol-red free cell culture medium to reduce adverse effects from keeping cells in 1 × PBS for an extended time and a sophisticated calibration of optical density^33^, may improve the reliability of the assay. Our assay measures integrated signals from an ensemble of cells rather than investigating individual cells, and therefore it cannot provide detailed information on the cellular uptake of nanoparticles unlike other techniques such as microscopy. Instead, it has an advantage of being able to quickly and simultaneously evaluate the uptake of nanoparticles of multiple samples under various conditions with an easily accessible setup consisting of routine cell culture and plate reading. Therefore, it can serve as a useful tool to complement existing sophisticated and precise nanoparticle uptake assays.

## Methods

### Cell culture

Jurkat, A549, and RPMI8226 cells were maintained in Roswell Park Memorial Institute (RPMI) 1640 medium supplemented with 10% fetal bovine serum, 200 units per mL penicillin, and 200 units per mL streptomycin, and incubated at 37 °C in a humidified 5% CO_2_ atmosphere. All cell lines were obtained from ATCC (American Type Culture Collection). Phase contrast images of the cells were obtained using an inverted microscope (Olympus IX71). All culture supplies were obtained from Thermo Fisher Scientific unless otherwise indicated. The cell counting and viability assay was performed with a Countess II FL using the trypan blue exclusion method (Thermo Fisher Scientific).

For the suspension cells, Jurkat and RPMI8226, cells were maintained not exceeding 1–2 × 10^6^ cells/mL and avoiding medium acidification, and an appropriate number of cells were prepared before the nanoparticle treatment. For the adherent cells, A549, cells were seeded in 6-well plates at a density of 2–3 × 10^5^ cells per well. The following day, the culture medium was replaced with fresh medium and used for the nanoparticle treatment. For a 96-well plate experiment, A549 cells were seeded in a 96-well plate at a density of 6–8 × 10^4^ cells per well. The following day, the culture medium was replaced with fresh medium and used for the nanoparticle treatment.

### Nanoparticle treatment and cell harvest

For the suspension cells, 4–5 × 10^5^ cells per well were seeded in a 6-well plate and then an appropriate volume of 10 × concentrated 50 nm green-dyed polystyrene nanoparticles (Fluoro-Max Dyed Green Aqueous Fluorescent Particle, excitation and emission peaks of 468 and 508 nm, Thermo Fisher Scientific) prepared in culture medium was added and mixed by pipetting. For the adhesion cells, an appropriate volume of 10 × concentrated 50 nm green-dyed polystyrene nanoparticles prepared in culture medium was added and mixed by gentle agitating by hand. For a routine assay, after 24 h, cells were harvested and washed twice with a sufficient volume of 1 × phosphate buffered saline (PBS) to remove residual nanoparticles both in the culture media and on the cell surfaces. The cells from each well were resuspended in 0.6 mL of 1 × PBS and kept at 4 °C before further analyses. For a kinetics assay, cells were harvested at 2, 4, 6, and 24 h and prepared for analyses. For the 96-well plate experiment using A549 cells, cells were gently washed twice with 300 µL of 1 × PBS avoiding cell detachment and then 180 µL of 1 × PBS was added.

For the wash test, Jurkat cells were prepared in a 100 mm culture dish at a density of 3–4 × 10^6^ cells per 10 mL of culture media containing 100 µg/mL nanoparticles. After 24 h, cells were harvested and washed a different number of times with a sufficient volume of 1 × PBS, and then resuspended in 1 × PBS and kept at 400A0°C before further analyses.

### Plate reader measurement

Nanoparticle-treated cells were aliquoted at 180 µL each into three wells of a black-walled 96-well plate (Corning). The measurement was carried out immediately after aliquoting using a Synergy HTX microplate reader (BioTek). The plate was shaken for 2 s before the measurement. The fluorescence was measured from the bottom with excitation and emission peaks of 485 and 528 nm. The absorbance was measured from 400 to 650 nm at 50 nm intervals. The acquisition settings including gain remained the same for all experiments.

### Flow cytometry measurement

Cells were analyzed using a FACSVerse (BD Biosciences). For nanoparticle uptake analysis, the live single-cell population was gated in a plot of forward scatter versus side scatter after excluding cell debris and doublets, and its FITC histogram was used for analysis. For cell counting, either TruCount tubes or liquid counting beads (BD Biosciences) were used. For the former, 300 µL of diluted cell solution was added to the tubes, and for the latter, 250 µL of diluted cell solution and 50 µL of bead solution were mixed before measurement. Cells were diluted appropriately so as not to dominate the acquisition event. The acquisition settings including the voltage of each channel remained the same for all experiments. For analysis, the counting bead and non-bead populations were gated in a plot of fluorescence (e.g., FITC or PerCP-Cy5.5) versus side scatter, and then from the non-bead population, the cell population was gated excluding debris in a plot of forward scatter versus side scatter. All data were analyzed using FlowJo (version 10.8, FlowJo LLC).

### Live imaging for cell proliferation analysis

A549 cells were seeded in clear-bottom 35 mm dishes (ibidi) at a density of 2 × 10^5^ per well and monitored for 72 h using a Lionheart FX Automated Microscope (Biotek). Images were taken every 2 h. Images from 16 fields of view were collected per dish and analyzed together.

### Nanoparticle characterization

The hydrodynamic size and zeta potential of the nanoparticles were determined using a Zetasizer Nano ZSP (Malvern Panalytical). Results are reported as the average of three runs. Scanning transmission electron microscopy (STEM) images were obtained with an annular STEM detector (aSTEM) using scanning electron microscopy (ZEISS GeminiSEM 500). Particle diameter analysis was performed with ImageJ using Otsu's thresholding. Only independently separated nanoparticles were selected and measured, and a total of 360 nanoparticles were analyzed for the measurement.

The FI of the nanoparticles was measured with a home-built TIRF microscope. A 488 nm beam was used to excite nanoparticles immobilized on the surface through non-specific binding. The fluorescence of the samples was collected through an objective lens (UPLSAPO 60XW, Olympus) and imaged with an electron-multiplying CCD (iXon Ultra 888, Andor Technology). Movie files (2−3 s long) were recorded at 100 ms per frame. The FI of single particles was extracted and analyzed using IDL (ITT Visual Information Solution) and MATLAB (MathWorks).

## Supplementary Information

Supplementary Information.

## Data Availability

All data generated or analysed during this study are included in this published article and its supplementary information files.
