# Supplementary material for: Quantifying fluorescent nanoparticle uptake in mammalian cells using a plate reader
Source: Sci Rep. 2022 Nov 23;12:20146. doi: 10.1038/s41598-022-24480-3 (PMC9684140; doi:10.1038/s41598-022-24480-3)
Supplement: Supplementary file 1 — Supplementary Information. [file 41598_2022_24480_MOESM1_ESM.pdf]

# Supplemental Data

## Quantifying fluorescent nanoparticle uptake in mammalian cells using a plate reader

Hye Ji Shin[1], Minjeong Kwak[2], Sihwa Joo[3], and Ji Youn Lee [1*]

[1]Biometrology Group, Division of Chemical and Biological Metrology, Korea Research Institute of Standards and Science, 267 Gajeong-ro, Yuseong-gu, Daejeon 34113, Republic of Korea

[2]Nanosafety Team, Safety Measurement Institute, Korea Research Institute of Standards and Science, 267 Gajeong-ro, Yuseong-gu, Daejeon 34113, Republic of Korea

[3]Bioimaging Team, Safety Measurement Institute, Korea Research Institute of Standards and Science, 267 Gajeong-ro, Yuseong-gu, Daejeon 34113, Republic of Korea

*Corresponding author

E-mail: jylee@kriss.re.kr (JYL)

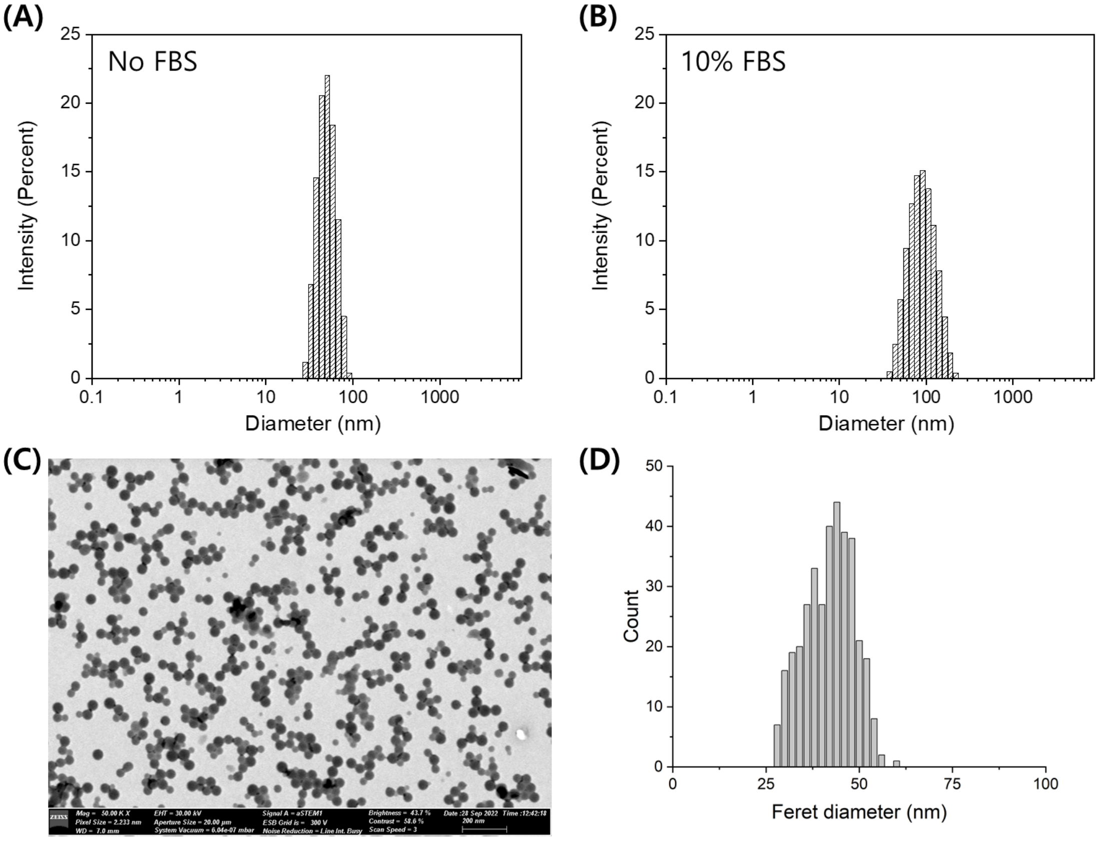


(E)

| Dispersant | | | 0% FBS media | 10% FBS media |
|---|---|---|---|---|
| **z-average*** | (d, nm) | | 46.23±0..13 | 83.68±0.76 |
| **SEM measurement** | (nm) | 40.07±6.46 | | |
| **Zeta potential*** | (mV) | | -47.30 ± 1.80 | -13.50 ± 0.70 |

* 100 μg/mL PSNP50

**Figure S1.** (A,B) Histogram plots showing the hydrodynamic size of PSNP50 in (A) basal media and (B) complete media containing 10% FBS measured via DLS. (C) STEM image of PSNP50 and (D) a histogram plot of ferret diameter. (E) Summary of size and zeta potential measurements of PSNP50.

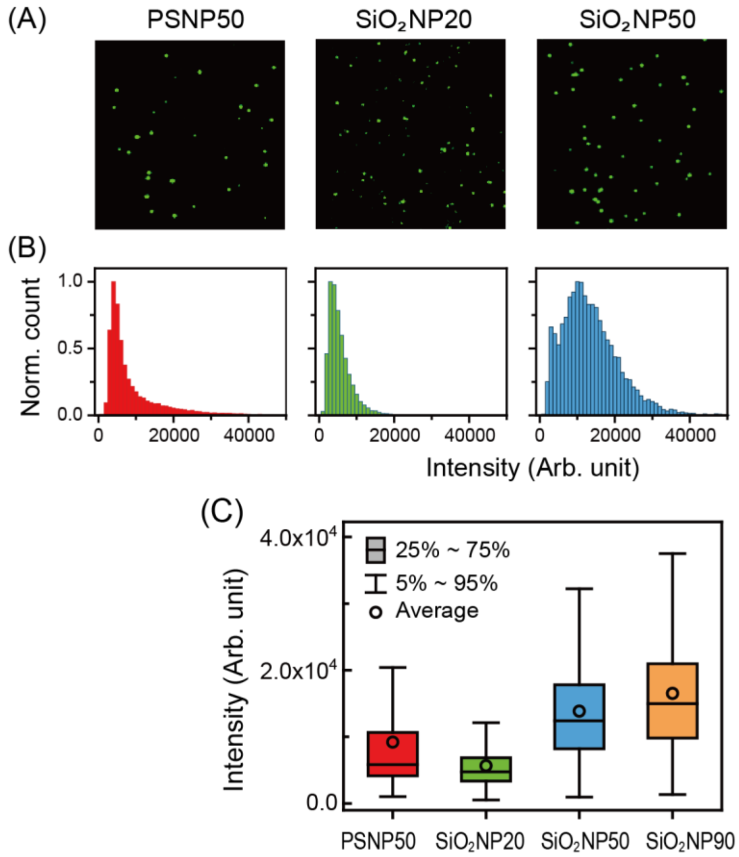


**Figure S2.** Fluorescence intensity (FI) of PSNP50 measured with TIRF microscopy. (A) Representative images of PSNP50 and 20, 50, and 90 nm FITC-labeled silica nanoparticles ($SiO_2$NP20, $SiO_2$NP50, and $SiO_2$NP90). (B) FI histograms of the single nanoparticles obtained from the analysis of TIRF images. (C) Graph comparing the FI of PSNP50 with FITC-labeled $SiO_2$NPs of different sizes. The PSNP50 was dimmer than the $SiO_2$NP of similar size ($SiO_2$NP50); however, its brightness was more uniform compared to $SiO_2$NP50 and $SiO_2$NP90.

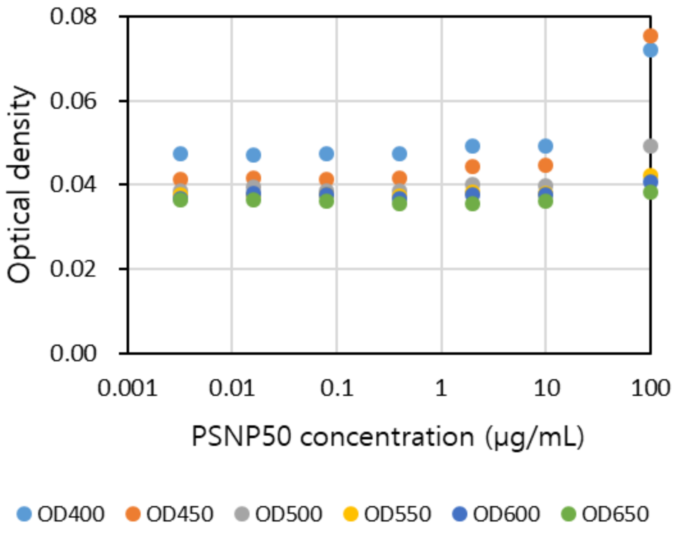


**Figure S3.** Scatter plots for absorbances of serially diluted PSNP50 ranging from 0.0032 to 100 µg/mL measured at various wavelengths.

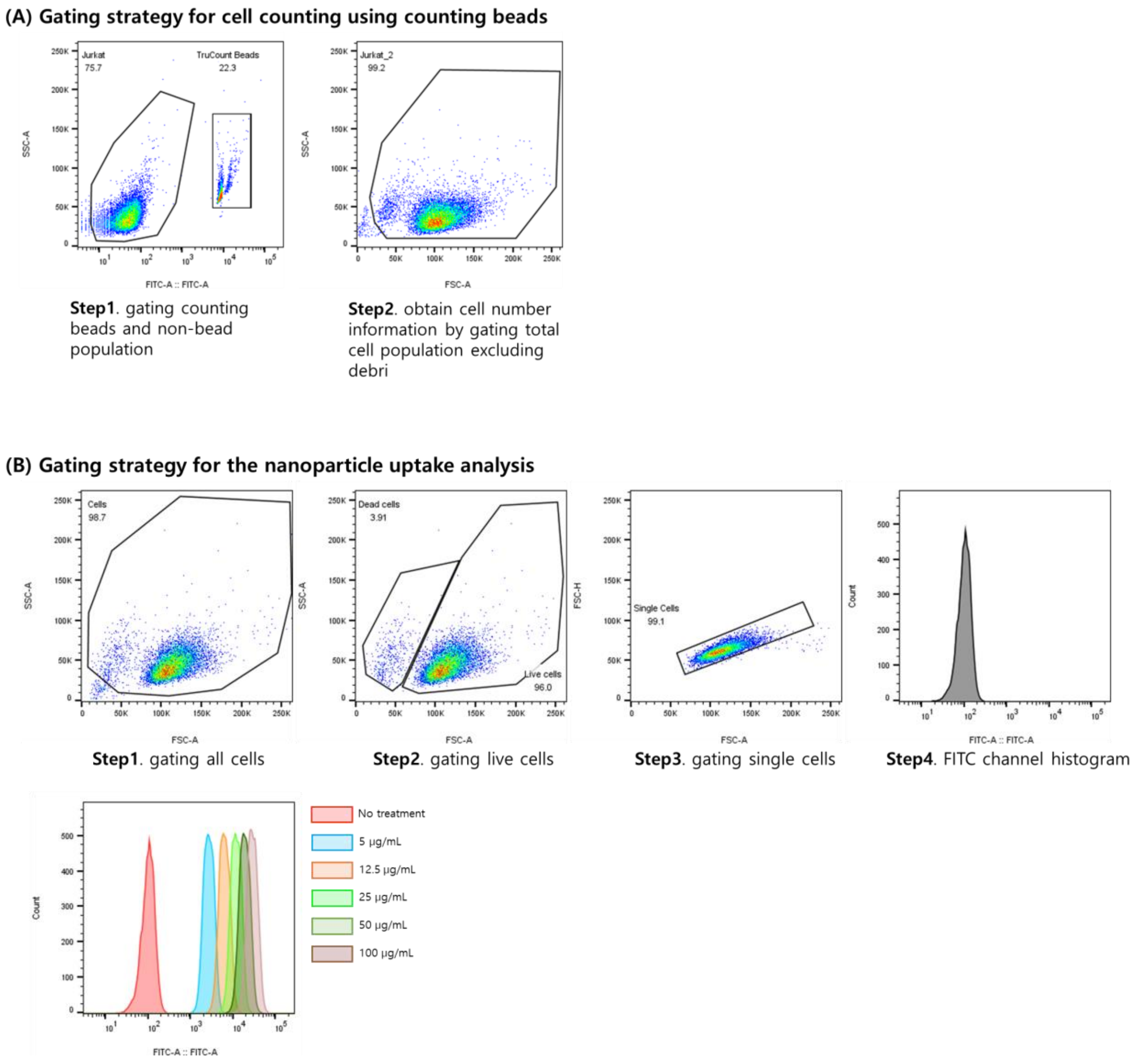


**Figure S4.** Gating strategies for flow cytometer analysis. **(A)** precise cell counting and **(B)** nanoparticle uptake analysis for Jurkat cells.

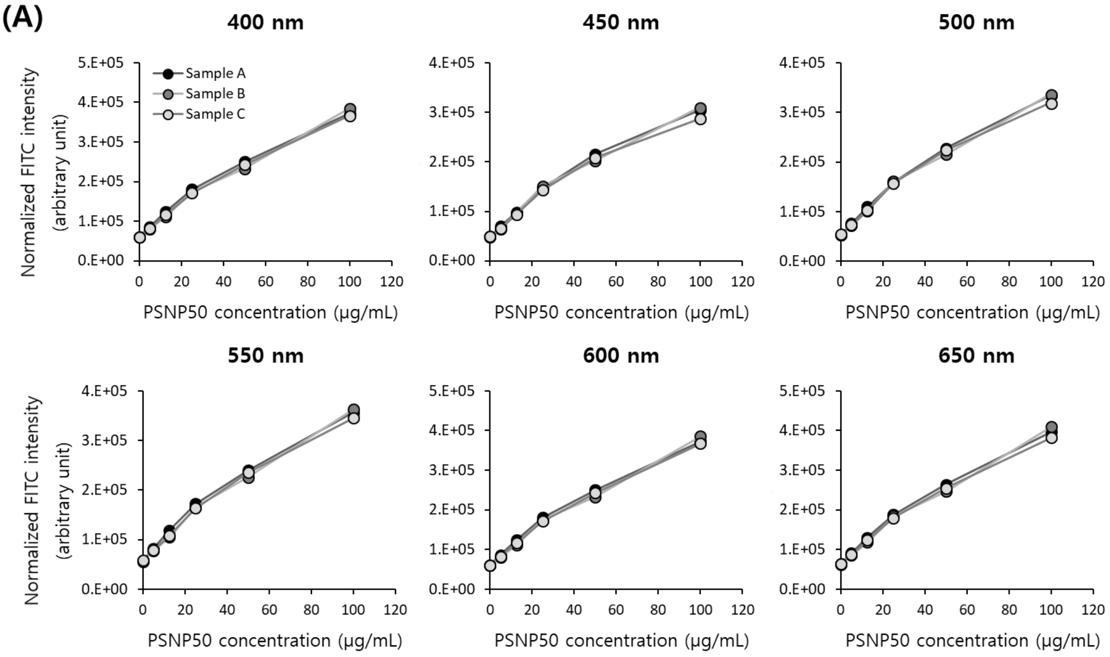


(B)

| FITC-PSNP50 (μg/mL) | Average | | | | | | CV | | | | | |
|---|---|---|---|---|---|---|---|---|---|---|---|---|
| | OD400 | OD450 | OD500 | OD550 | OD600 | OD650 | OD400 | OD450 | OD500 | OD550 | OD600 | OD650 |
| 0 | 100% | 101% | 101% | 101% | 100% | 99% | 0.40% | 1.16% | 1.88% | 2.74% | - | 1.62% |
| 5 | 95% | 96% | 97% | 98% | 100% | 97% | 4.87% | 4.06% | 2.89% | 2.85% | - | 2.84% |
| 12.5 | 97% | 97% | 96% | 95% | 100% | 95% | 2.11% | 2.40% | 3.97% | 6.32% | - | 4.69% |
| 25 | 101% | 101% | 100% | 98% | 100% | 96% | 4.60% | 2.88% | 1.21% | 2.72% | - | 2.35% |
| 50 | 105% | 104% | 101% | 99% | 100% | 96% | 2.47% | 3.35% | 2.91% | 2.92% | - | 3.26% |
| 100 | 98% | 101% | 101% | 101% | 100% | 100% | 3.66% | 4.01% | 2.94% | 2.51% | - | 3.48% |

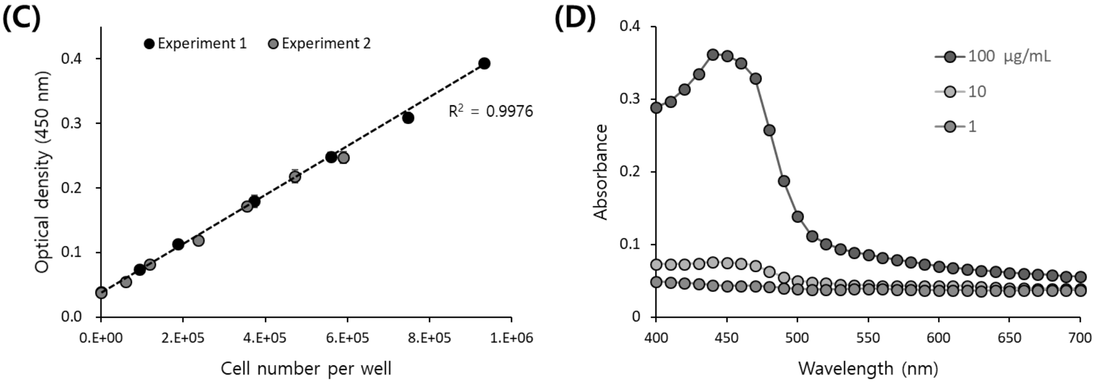


**Figure S5.** Nanoparticle uptake assay results of Jurkat cells by normalizing FITC intensities with ODs of different wavelengths. (A) Normalized FITC intensity graphs and (B) relative normalized FITC intensities to OD600 results shows that the results are comparable between analyses. (C) Correlation between cell number and optical density measured at 450 nm. (D) Absorbance spectrum of PSNP50 nanoparticles.

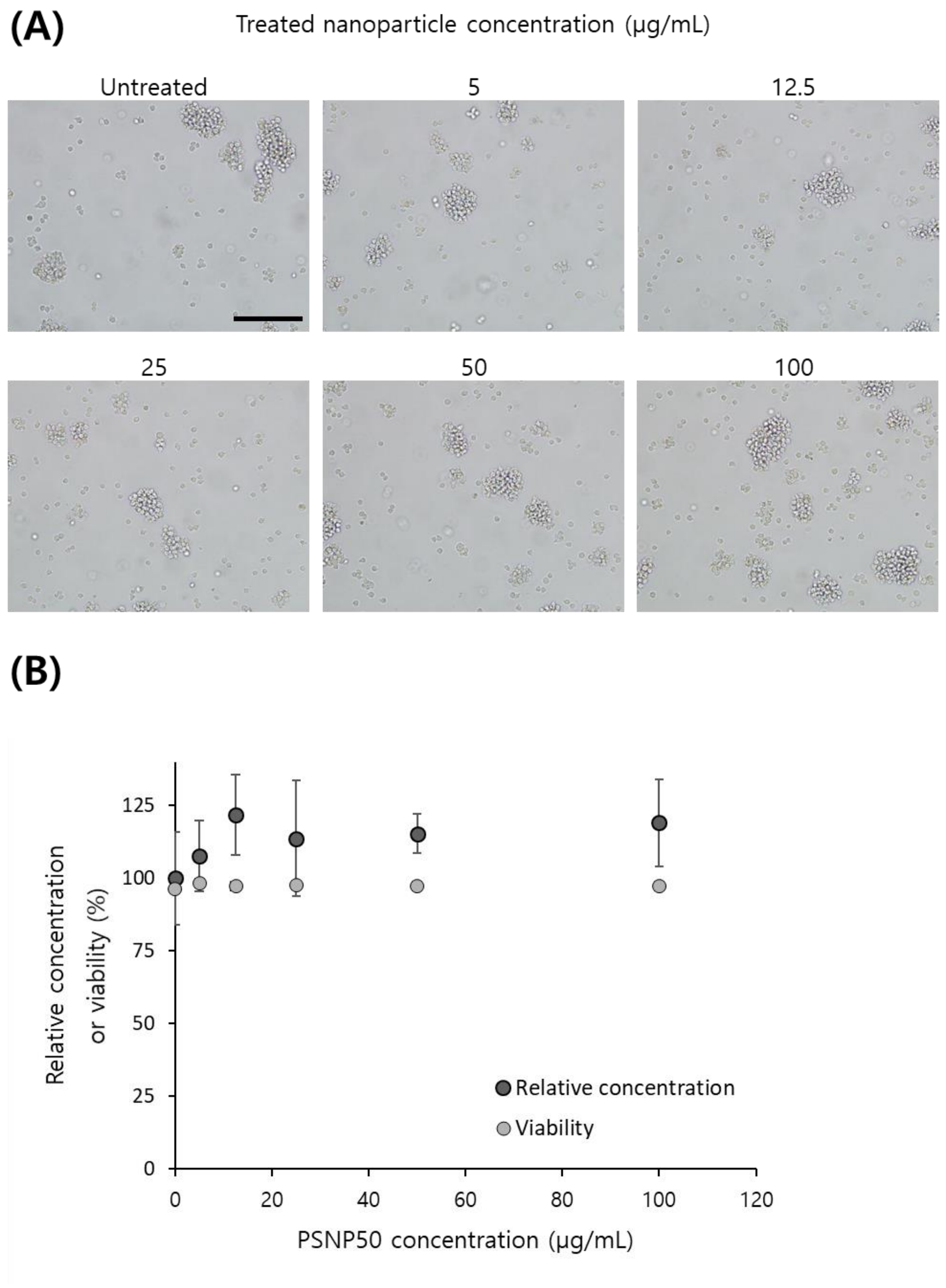


**Figure S6.** Effect of the presence of PSNP50 on the **(A)** morphology and **(B)** viability and relative concentration of Jurkat cells. Scale bar = 200 μm.

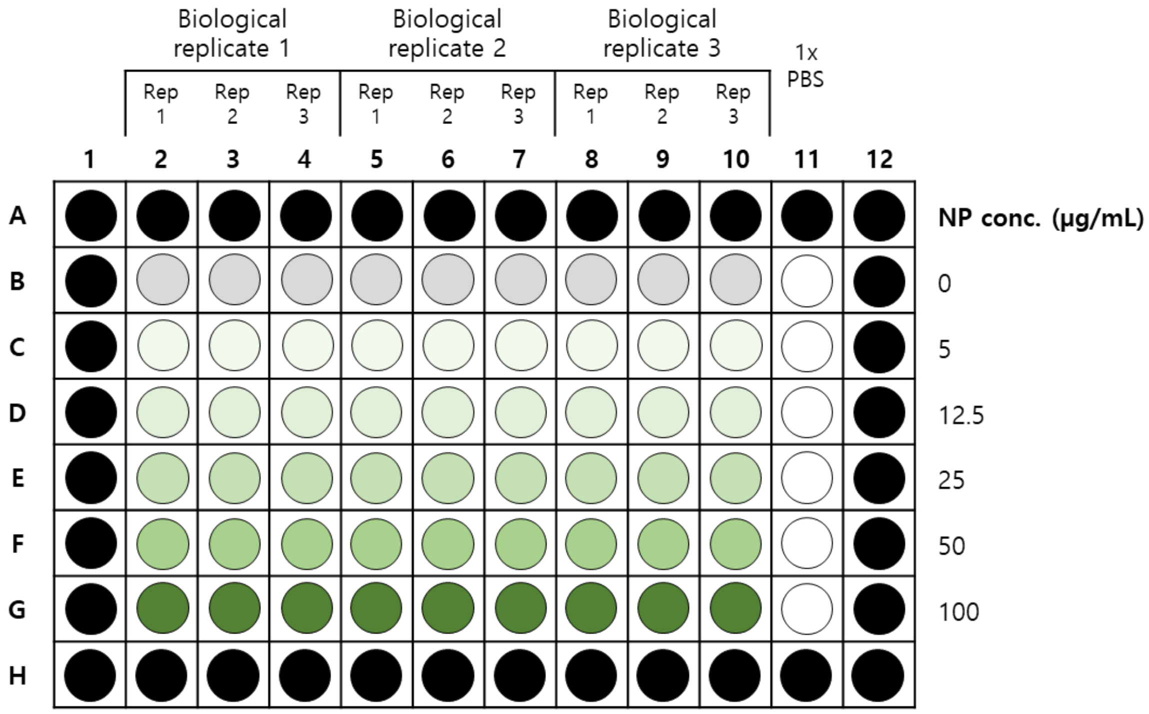


**Figure S7.** Sample layout in a 96-well plate for plate reading.

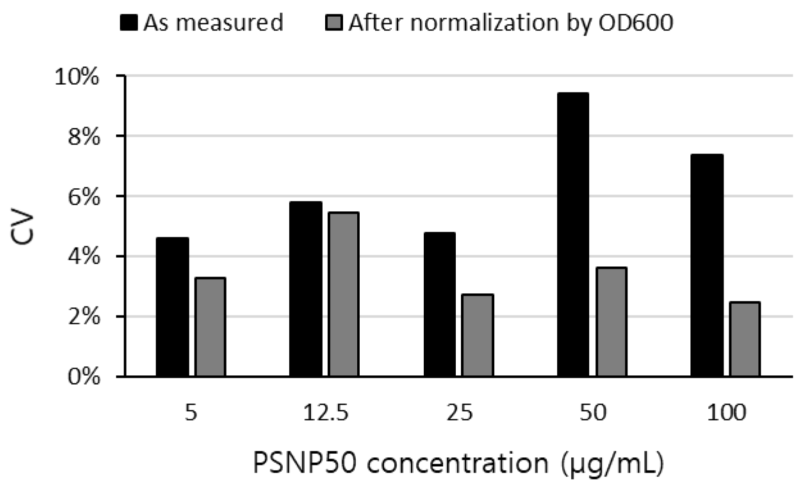


**Figure S8.** Change in CV of FITC intensity of nanoparticle-treated Jurkat cells of triplicates by normalization with absorbance at 600 nm.

(A)

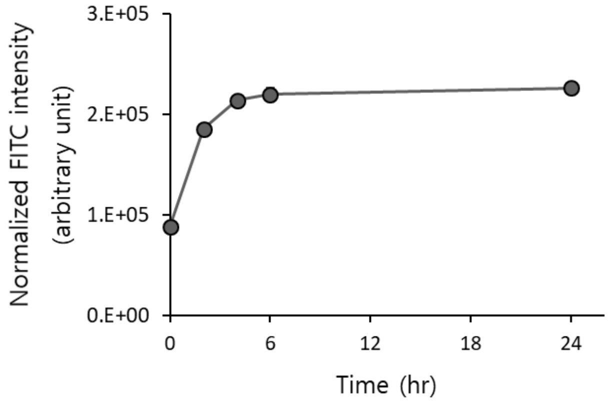


(B)

**Measured fluorescence intensity**

| Time (hr) | Sample 1 | | | Sample 2 | | |
|---|---|---|---|---|---|---|
| 0 | 5944 | 5966 | 6013 | 5995 | 6030 | 6026 |
| 2 | 13328 | 13856 | 13768 | 11759 | 11417 | 11652 |
| 4 | 12402 | 14969 | 12729 | 13939 | 14035 | 13647 |
| 6 | 15544 | 15374 | 15366 | 15310 | 15278 | 15120 |
| 24 | 25429 | 25598 | 25429 | 23348 | 23109 | 23144 |

**OD600**

| Time (hr) | Sample 1 | | | Sample 2 | | |
|---|---|---|---|---|---|---|
| 0 | 0.069 | 0.07 | 0.071 | 0.066 | 0.065 | 0.064 |
| 2 | 0.073 | 0.071 | 0.073 | 0.068 | 0.06 | 0.063 |
| 4 | 0.057 | 0.06 | 0.059 | 0.063 | 0.06 | 0.06 |
| 6 | 0.064 | 0.064 | 0.067 | 0.067 | 0.068 | 0.068 |
| 24 | 0.105 | 0.108 | 0.106 | 0.098 | 0.097 | 0.098 |

**Normalized fluorescence intensity**

| Time (hr) | Sample 1 | | | Sample 2 | | | Average | | | | |
|---|---|---|---|---|---|---|---|---|---|---|---|
| | | | | | | | Sample 1 | Sample 2 | Average | Std. Dev. | RSD (%) |
| 0 | 86145 | 85229 | 84690 | 90833 | 92769 | 94156 | 85355 | 92586 | **88970** | **5114** | 5.75 |
| 2 | 182575 | 195155 | 188603 | 172926 | 190283 | 184952 | 188778 | 182721 | **185749** | **4283** | 2.31 |
| 4 | 206700 | 237603 | 205306 | 211197 | 215923 | 206773 | 216537 | 211298 | **213917** | **3704** | 1.73 |
| 6 | 228588 | 229463 | 216423 | 215634 | 215183 | 216000 | 224824 | 215606 | **220215** | **6519** | 2.96 |
| 24 | 225035 | 224544 | 227045 | 226680 | 224359 | 231440 | 225541 | 227493 | **226517** | **1380** | 0.61 |

**Figure S9.** Nanoparticle uptake dynamics of Jurkat cells measured for 24 h. (A) Time-course graph and (B) corresponding raw data.

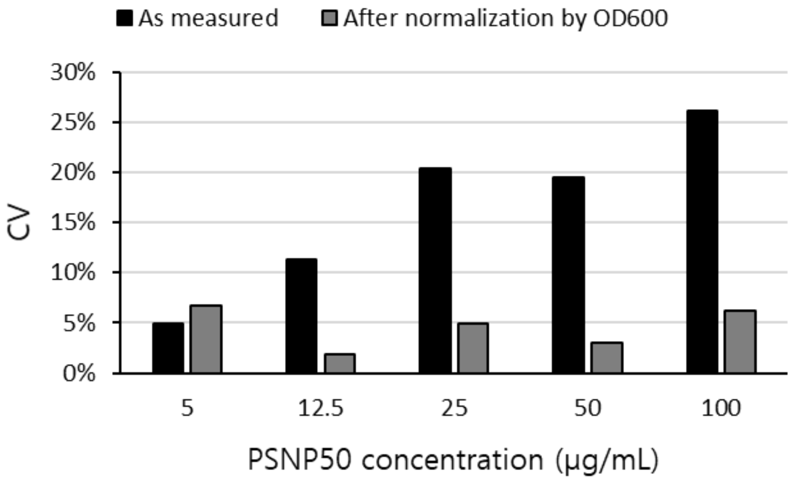


**Figure S10.** Change of CV in FITC intensity of nanoparticle-treated Jurkat cells of three sets of experiments (displayed in Figure 4 in the main text) by normalization to absorbance at 600 nm.

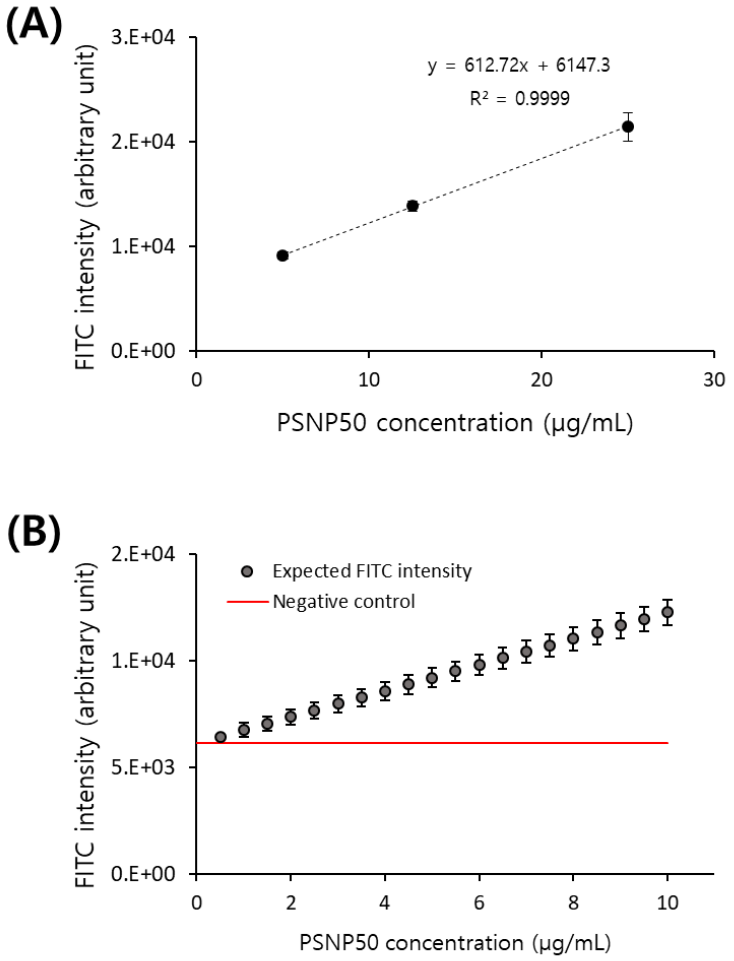


**Figure S11.** Estimation of the limit of quantification of the nanoparticle uptake assay. (A) Plot between nanoparticle concentration and measured fluorescence intensity showing a high correlation especially in the lower concentration range. (B) Simulated fluorescence intensities of nanoparticles with concentrations lower than 10 μg/mL.

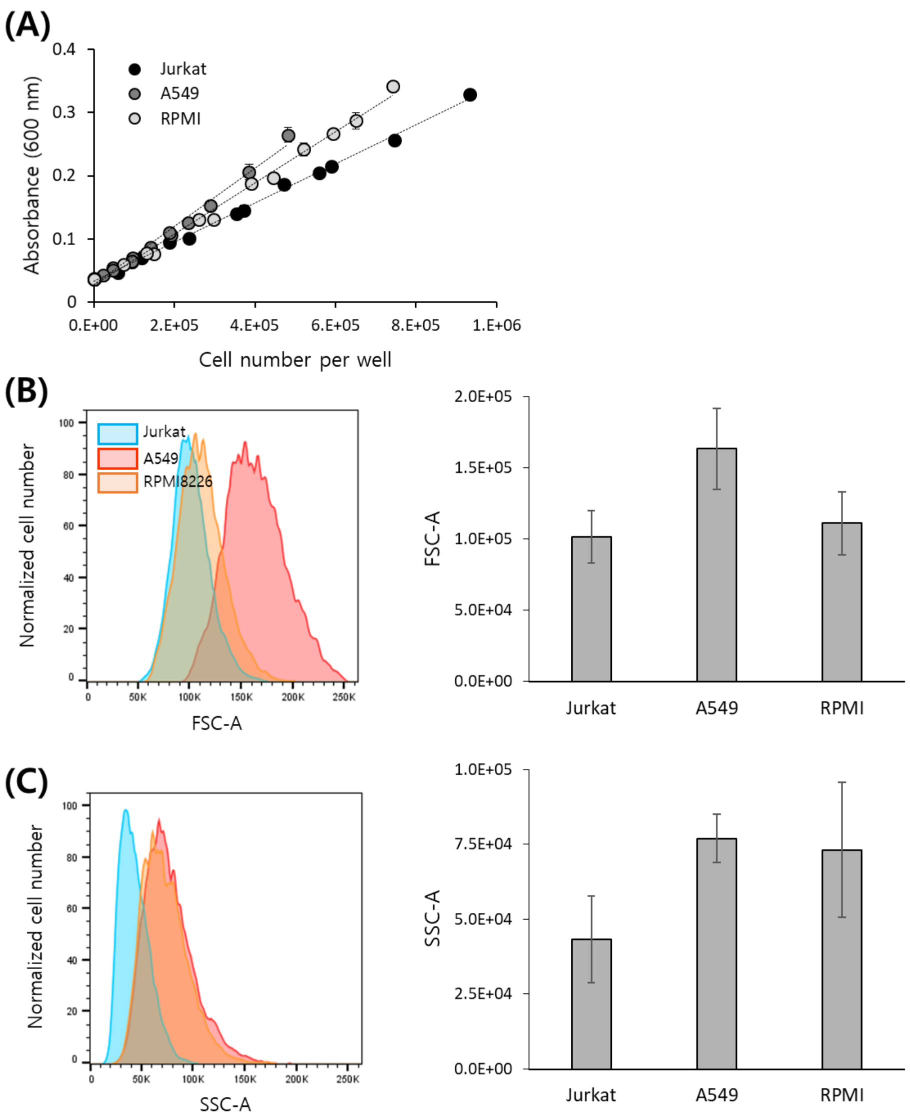


**Figure S12.** Comparison of three cell lines in absorbance and scattering. **(A)** Correlation between cell number and optical density measured at 600 nm, **(B)** FSC intensity histogram and its mean value, **(C)** FSC intensity histogram and its mean value for each cell line.

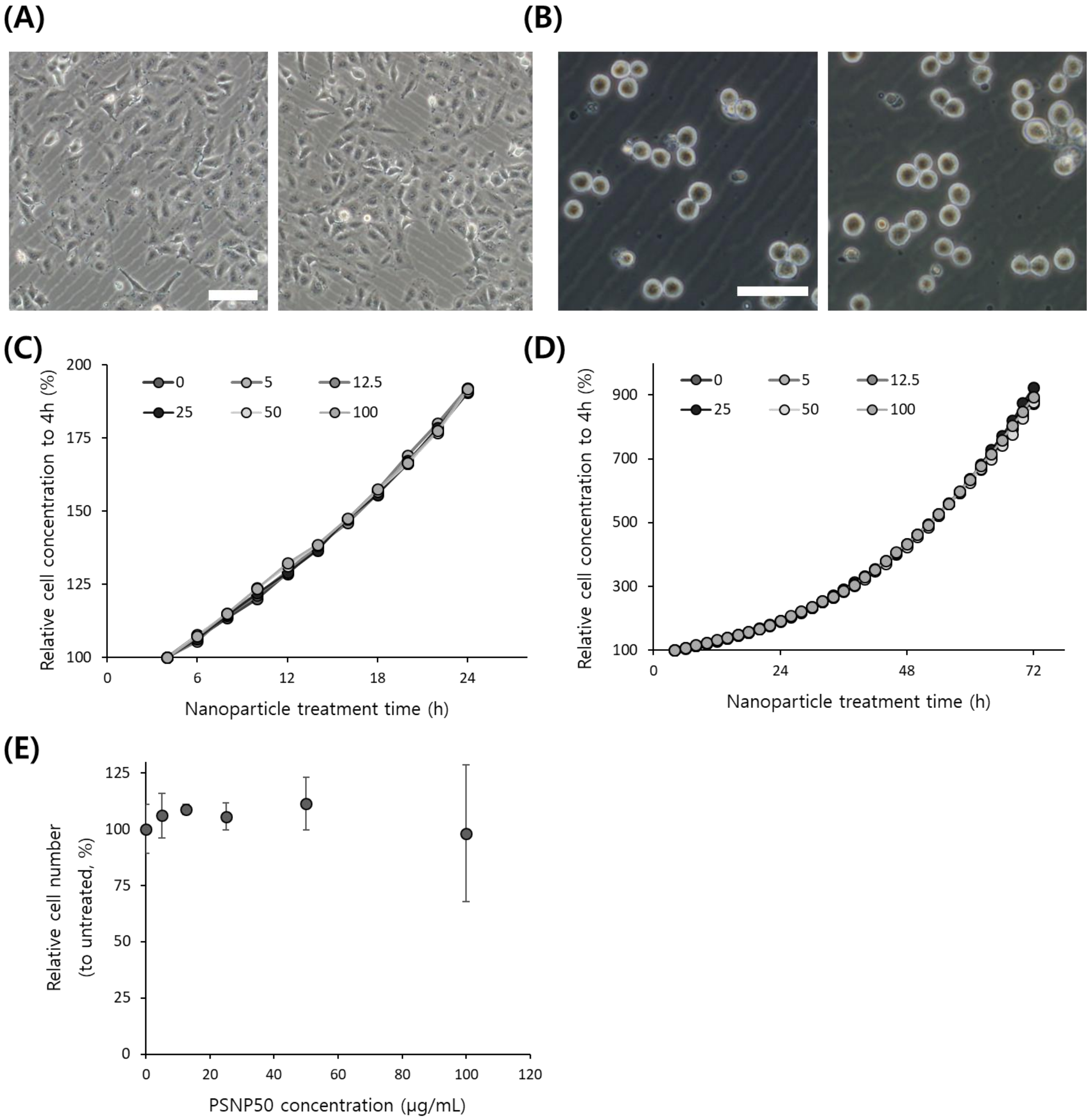


**Figure S13.** Effect of the presence of PSNP50 on the cell morphology and proliferation of A549 and RPMI8226 cells. **(A)** Untreated (left panel) and 100 μg/mL nanoparticle treated (right panel) A549 cells after 24 h. **(B)** Untreated (left panel) and 100 μg/mL nanoparticle treated (right panel) RPMI8226 cells after 24 h. **(C), (D)** Cell growth profile of A549 cells measured by live cell imaging after nanoparticle treatment for 24 h and 72 h. **(E)** Relative cell number of RPMI8226 cells after 24 h nanoparticle treatment. Scale bars = 100 μm.

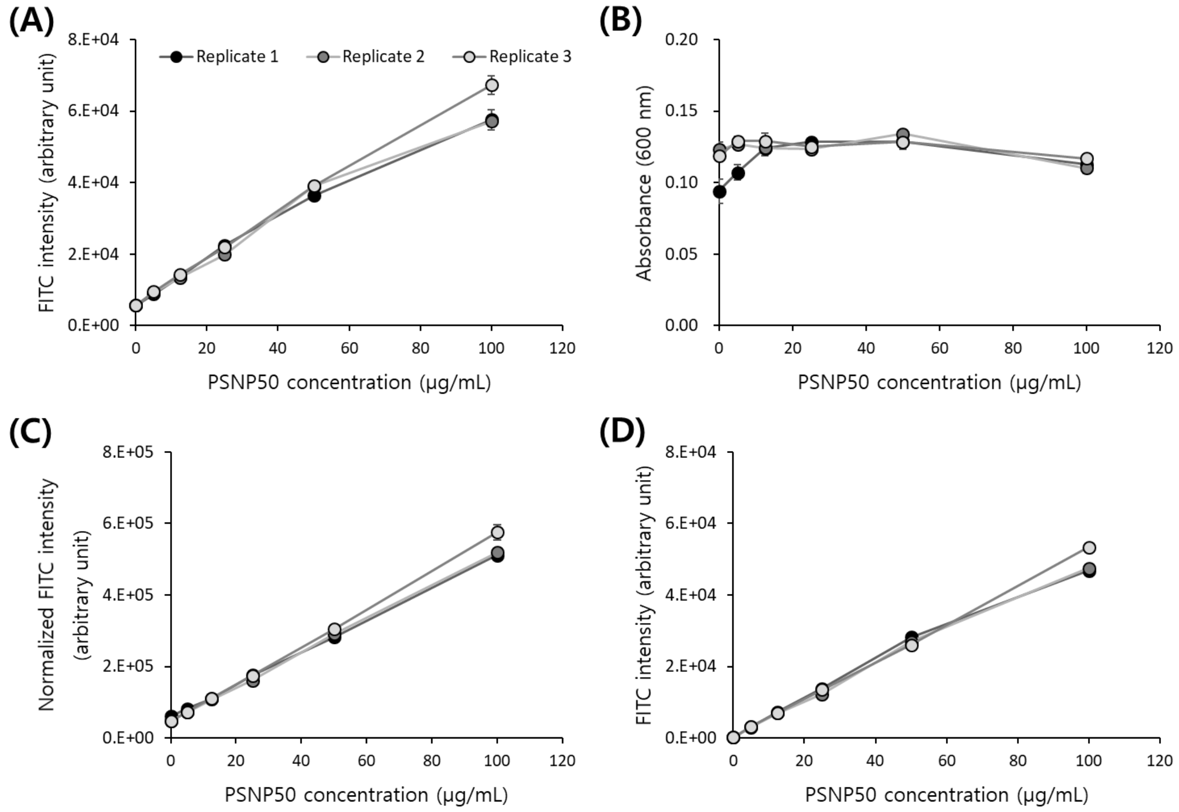


**Figure S14.** Quantification of nanoparticle uptake by A549 cells. Scatter plots for **(A)** FITC intensity measured by a plate reader, **(B)** optical density measured at 600 nm, **(C)** normalized FITC intensity by the optical density, and **(D)** FITC intensity measured by a flow cytometer of three technical replicates of A549 cells treated with different concentrations of PSNP50.

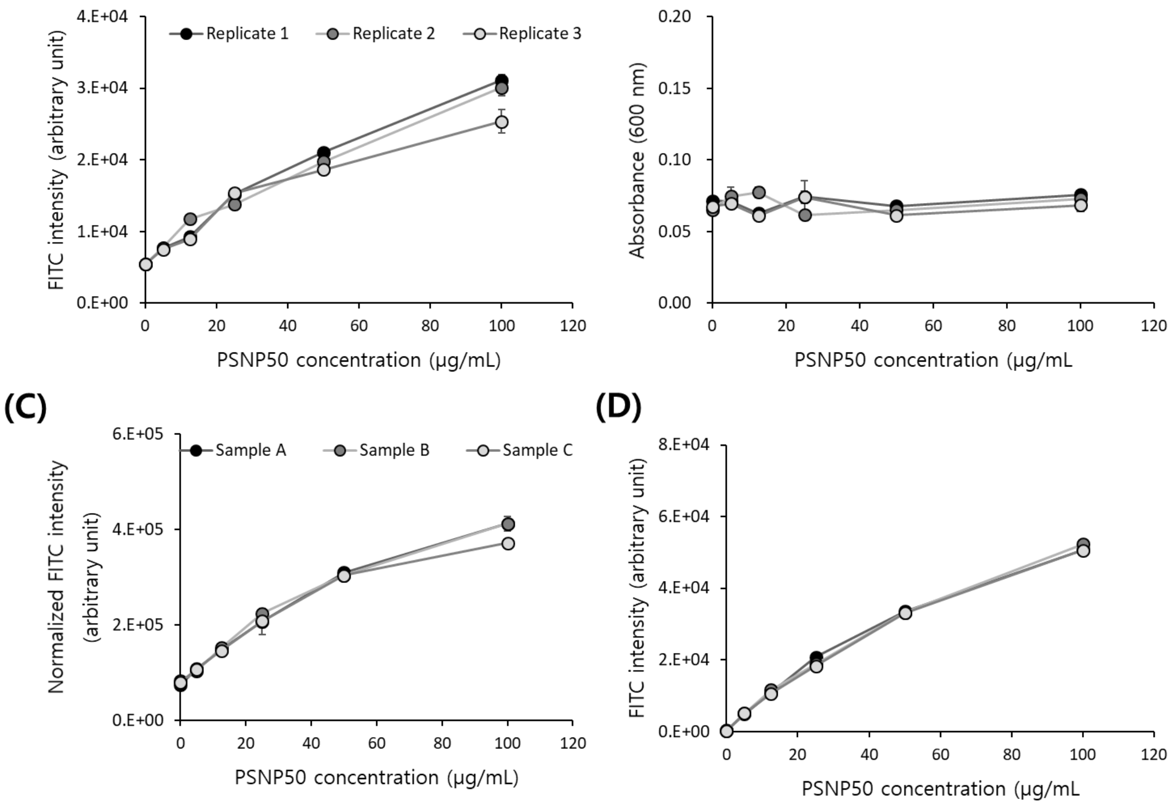


**Figure S15.** Quantification of nanoparticle uptake by RPMI8226 cells. Scatter plots for **(A)** FITC intensity measured by a plate reader, **(B)** optical density measured at 600 nm, **(C)** normalized FITC intensity by the optical density, and **(D)** FITC intensity measured by a flow cytometer of three technical replicates of RPMI8226 cells treated with different concentrations of PSNP50.

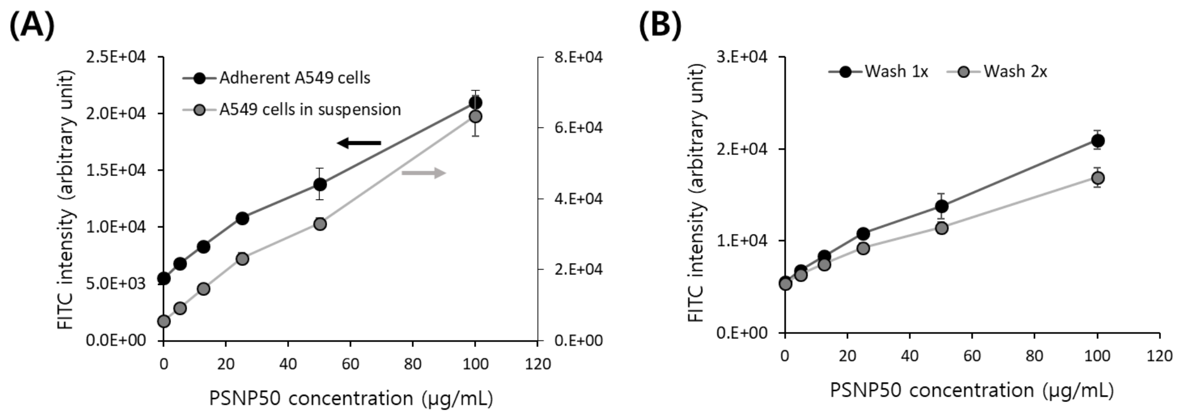


**Figure S16. (A)** Comparison of nanoparticle uptake assays of A549 cells in adherent (single 96-well plate) and suspension format. **(B)** Effect of number of washings on the FITC intensity of A549 cells in a single 96-well plate format.

**Table S1.** Raw and analyzed data for the scatter plots of Figure 3 (main text).

Microplate reader: FITC channel

| | Replicate 1 | | | Replicate 2 | | | Replicate 3 | | | Average | | | Std. Dev. | | | RSD | | | Between Replicates | | |
|---|---|---|---|---|---|---|---|---|---|---|---|---|---|---|---|---|---|---|---|---|---|
| PSNP50 (µg/mL) | Well 1 | Well 2 | Well 3 | Well 1 | Well 2 | Well 3 | Well 1 | Well 2 | Well 3 | Replicate 1 | Replicate 2 | Replicate 3 | Replicate 1 | Replicate 2 | Replicate 3 | Replicate 1 | Replicate 2 | Replicate 3 | Average | Std. Dev. | RSD |
| 0 | 5435 | 5532 | 5480 | 5519 | 5472 | 5472 | 5489 | 5487 | 5469 | 5482 | 5488 | 5482 | 49 | 27 | 11 | 0.89% | 0.49% | 0.20% | 5484 | 3 | 0.06% |
| 5 | 7700 | 7661 | 7739 | 8277 | 8309 | 8321 | 8499 | 8414 | 8254 | 7700 | 8302 | 8389 | 39 | 23 | 124 | 0.51% | 0.27% | 1.48% | 8130 | 375 | 4.62% |
| 12.5 | 10739 | 11170 | 11347 | 11709 | 11569 | 11554 | 12607 | 12512 | 12170 | 11085 | 11611 | 12430 | 313 | 85 | 230 | 2.82% | 0.74% | 1.85% | 11709 | 677 | 5.79% |
| 25 | 16590 | 16949 | 17012 | 16731 | 17205 | 17097 | 18192 | 18497 | 18393 | 16850 | 17011 | 18361 | 228 | 248 | 155 | 1.35% | 1.46% | 0.84% | 17407 | 830 | 4.77% |
| 50 | 21594 | 20775 | 22916 | 25554 | 24922 | 24719 | 27121 | 25700 | 25658 | 21762 | 25065 | 26160 | 1080 | 435 | 833 | 4.96% | 1.74% | 3.18% | 24329 | 2290 | 9.41% |
| 100 | 28656 | 30292 | 30275 | 33379 | 33765 | 32674 | 29314 | 28829 | 28478 | 29741 | 33273 | 28874 | 940 | 553 | 420 | 3.16% | 1.66% | 1.45% | 31507 | 2330 | 7.40% |

Microplate reader: Absorbance at 600 nm

| | Replicate 1 | | | Replicate 2 | | | Replicate 3 | | | Average | | | Std. Dev. | | | RSD | | | Between samples | | |
|---|---|---|---|---|---|---|---|---|---|---|---|---|---|---|---|---|---|---|---|---|---|
| PSNP50 (µg/mL) | Well 1 | Well 2 | Well 3 | Well 1 | Well 2 | Well 3 | Well 1 | Well 2 | Well 3 | Sample A | Sample B | Sample C | Sample A | Sample B | Sample C | Replicate 1 | Replicate 2 | Replicate 3 | Average | Std. Dev. | RSD |
| 0 | 0.095 | 0.092 | 0.087 | 0.091 | 0.094 | 0.090 | 0.086 | 0.095 | 0.090 | 0.091 | 0.092 | 0.090 | 0.004 | 0.002 | 0.005 | 4.42% | 2.27% | 4.99% | 0.091 | 0.001 | 0.76% |
| 5 | 0.095 | 0.086 | 0.088 | 0.102 | 0.103 | 0.104 | 0.104 | 0.101 | 0.100 | 0.090 | 0.103 | 0.102 | 0.005 | 0.001 | 0.002 | 5.27% | 0.97% | 2.05% | 0.098 | 0.007 | 7.48% |
| 12.5 | 0.087 | 0.093 | 0.089 | 0.102 | 0.106 | 0.106 | 0.112 | 0.108 | 0.102 | 0.090 | 0.105 | 0.107 | 0.003 | 0.002 | 0.005 | 3.41% | 2.21% | 4.69% | 0.101 | 0.010 | 9.47% |
| 25 | 0.092 | 0.097 | 0.091 | 0.094 | 0.096 | 0.104 | 0.109 | 0.112 | 0.101 | 0.093 | 0.098 | 0.107 | 0.003 | 0.005 | 0.006 | 3.44% | 5.40% | 5.30% | 0.100 | 0.007 | 7.16% |
| 50 | 0.086 | 0.076 | 0.101 | 0.112 | 0.107 | 0.104 | 0.113 | 0.104 | 0.106 | 0.088 | 0.108 | 0.108 | 0.013 | 0.004 | 0.005 | 14.35% | 3.75% | 4.39% | 0.101 | 0.012 | 11.43% |
| 100 | 0.078 | 0.081 | 0.080 | 0.086 | 0.087 | 0.086 | 0.079 | 0.078 | 0.079 | 0.080 | 0.086 | 0.079 | 0.002 | 0.001 | 0.001 | 1.92% | 0.67% | 0.73% | 0.082 | 0.004 | 5.11% |

After normalization by OD600

| | Replicate 1 | | | Replicate 2 | | | Replicate 3 | | | Average | | | Std. Dev. | | | RSD | | | Between Replicates | | |
|---|---|---|---|---|---|---|---|---|---|---|---|---|---|---|---|---|---|---|---|---|---|
| PSNP50 (µg/mL) | Well 1 | Well 2 | Well 3 | Well 1 | Well 2 | Well 3 | Well 1 | Well 2 | Well 3 | Replicate 1 | Replicate 2 | Replicate 3 | Replicate 1 | Replicate 2 | Replicate 3 | Replicate 1 | Replicate 2 | Replicate 3 | Average | Std. Dev. | RSD |
| 0 | 57211 | 60130 | 62989 | 60648 | 58213 | 60800 | 63826 | 57758 | 60767 | 60110 | 59887 | 60783 | 2889 | 1452 | 3034 | 4.81% | 2.42% | 4.99% | 60260 | 467 | 0.77% |
| 5 | 81053 | 89081 | 87943 | 81147 | 80670 | 80010 | 81721 | 83307 | 82540 | 86026 | 80609 | 82523 | 4344 | 571 | 793 | 5.05% | 0.71% | 0.96% | 83052 | 2747 | 3.31% |
| 12.5 | 123437 | 120108 | 127494 | 114794 | 109142 | 109000 | 112563 | 115852 | 119314 | 123680 | 110979 | 115909 | 3699 | 3305 | 3376 | 2.99% | 2.98% | 2.91% | 116856 | 6403 | 5.48% |
| 25 | 180326 | 174732 | 186945 | 177989 | 179219 | 164394 | 166899 | 165152 | 182109 | 180668 | 173867 | 171387 | 6114 | 8227 | 9327 | 3.38% | 4.73% | 5.44% | 175307 | 4805 | 2.74% |
| 50 | 251093 | 273355 | 226891 | 228161 | 232916 | 237683 | 240009 | 247115 | 242057 | 250446 | 232920 | 243060 | 23239 | 4761 | 3658 | 9.28% | 2.04% | 1.50% | 242142 | 8799 | 3.63% |
| 100 | 367385 | 373975 | 378438 | 388128 | 388103 | 379930 | 371063 | 369603 | 360481 | 373266 | 385387 | 367049 | 5560 | 4726 | 5735 | 1.49% | 1.23% | 1.56% | 375234 | 9326 | 2.49% |

Flow cytometer data (median)

| PSNP50 (µg/mL) | Replicate 1 | Replicate 2 | Replicate 3 | Average | Std. Dev. | RSD |
|---|---|---|---|---|---|---|
| 0 | 74.5 | 68.1 | 57.8 | 67 | 8 | 12.61% |
| 5 | 2346 | 2056 | 2568 | 2323 | 257 | 11.05% |
| 12.5 | 5688 | 5762 | 5989 | 5813 | 157 | 2.70% |
| 25 | 10626 | 10970 | 11197 | 10931 | 287 | 2.63% |
| 50 | 17820 | 17737 | 17988 | 17848 | 128 | 0.72% |
| 100 | 27476 | 27872 | 26828 | 27392 | 527 | 1.92% |
